# Editorial: Malaria in East African Highlands: Impact of Environmental Changes

**DOI:** 10.3389/fpubh.2017.00198

**Published:** 2017-08-02

**Authors:** Yousif El Safi Himeidan

**Affiliations:** ^1^Africa Technical Research Centre, Vector Health International Ltd., Arusha, Tanzania

**Keywords:** malaria, highlands, environmental changes, deforestation, long-lasting insecticidal nets

**Editorial on the Research Topic**

**Malaria in East African Highlands: Impact of Environmental Changes**

The current Research Topic “Malaria in East African Highlands: Impact of Environmental Changes” emerged from the article published in *Frontiers in Physiology* by Himeidan and Kweka. The authors documented that the demographic pressure of poor populations in the region (1–3) resulted in the extensive unprecedented environmental changes in the past 30 years (Himeidan and Kweka). These include land use and land cover changes such as modification of bush-land, woodland, and grassland on hillsides to farmland and transformation of papyrus swamps in valley bottoms to dairy pastures and cropland (Himeidan and Kweka). The loss of indigenous forests was also huge (4, 5). Previous experimental studies suggested that the unmitigated environmental changes in the highlands led to the rise in temperature hence optimizing the spread of favorable larval habitats (6), survival of adult malaria vectors (7), and development of malaria parasites (8).

The articles in the current assembled research topic gathered entomological and parasitological data from longitudinal active surveys conducted during the last 15 years in the East African highlands. The studies applied different methodological and analytical approaches and were able to establish an advanced understanding and documentation of the role of the environmental changes in malaria transmission and its distribution. Deforestation and replacement of natural swamp vegetation with agricultural crops were the key drivers for the temperature rise in indoor environment (microclimatic condition) and creation of suitable breeding habitats in open places [Kulkarni et al.; Kweka et al.; (7)]. Historically, malaria in the highlands is primarily affected by low ambient temperature and hence the increase in the temperature was significantly associated with the increase in the number of malaria vectors per house, enhancement of sporogony development rate, and survival of adult vectors leading to a higher risk of malaria transmission [Kweka et al.; (9–11)]. These environmental changes interrelate to the complex ecosystem comprising hills, plateaus, valleys, rivers, streams, and swamps that form clear profound effects on the level and distribution of malaria transmission (12–14). This confirmed the need for mapping this ecosystem to identify the affected areas for targeted vector control interventions including long-lasting insecticidal nets (LLINs), indoor residual spray (IRS), and larval control [Wanjala and Kweka; (15)].

Although the universal coverage of LLINs as recommended by WHO has already been achieved, new challenges have arisen including change in vector biting behavior, species composition, and increasing insecticide resistance as a consequence of both environmental changes and longitudinal use of LLINs [Zhou et al.; (16, 17)]. Despite these challenges, LLINs have been shown to be still effective in the highlands and have a significant community-wide benefit on reducing the overall parasite prevalence due to the significant suppression of the predominant vector, *Anopheles gambiae s.s*. (Zhou et al.). However, there is a concern about the control of the increasing outdoor malaria transmission in the highlands because of the resurgence of the other main malaria vectors in Africa, i.e., *Anopheles arabiensis* and *Anopheles funestus* [Zhou et al.; (17, 18)]. Without enhancing vector-control interventions, the ongoing environmental changes and insecticide resistance will continue to create a conducive condition for increasing malaria transmission in the highlands. In response to the wide use of a single class of insecticide in LLINs, target use of larval control, and IRS or using new LLINs with different insecticides as additional interventions are necessary to mitigate malaria resurgence in East African highlands.

## Author Contributions

The author listed has entirely made all direct and intellectual contributions to the work and approved it for publication.

## Conflict of Interest Statement

The author declares that the research was conducted in the absence of any commercial or financial relationships that could be construed as a potential conflict of interest.
